# Modelling the acid/base ^1^H NMR chemical shift limits of metabolites in human urine

**DOI:** 10.1007/s11306-016-1101-y

**Published:** 2016-09-15

**Authors:** Gregory D. Tredwell, Jacob G. Bundy, Maria De Iorio, Timothy M. D. Ebbels

**Affiliations:** 1 Department of Surgery and Cancer, Imperial College London, London, UK; 2 Department of Statistical Science, University College London, London, UK; 3 Department of Applied Mathematics, Australian National University, Canberra, Australia

**Keywords:** NMR, pH, Peak shift, Acid/base limits

## Abstract

**Introduction:**

Despite the use of buffering agents the ^1^H NMR spectra of biofluid samples in metabolic profiling investigations typically suffer from extensive peak frequency shifting between spectra. These chemical shift changes are mainly due to differences in pH and divalent metal ion concentrations between the samples. This frequency shifting results in a correspondence problem: it can be hard to register the same peak as belonging to the same molecule across multiple samples. The problem is especially acute for urine, which can have a wide range of ionic concentrations between different samples.

**Objectives:**

To investigate the acid, base and metal ion dependent ^1^H NMR chemical shift variations and limits of the main metabolites in a complex biological mixture.

**Methods:**

Urine samples from five different individuals were collected and pooled, and pre-treated with Chelex-100 ion exchange resin. Urine samples were either treated with either HCl or NaOH, or were supplemented with various concentrations of CaCl_2_, MgCl_2_, NaCl or KCl, and their ^1^H NMR spectra were acquired.

**Results:**

Nonlinear fitting was used to derive acid dissociation constants and acid and base chemical shift limits for peaks from 33 identified metabolites. Peak pH titration curves for a further 65 unidentified peaks were also obtained for future reference. Furthermore, the peak variations induced by the main metal ions present in urine, Na^+^, K^+^, Ca^2+^ and Mg^2+^, were also measured.

**Conclusion:**

These data will be a valuable resource for ^1^H NMR metabolite profiling experiments and for the development of automated metabolite alignment and identification algorithms for ^1^H NMR spectra.

**Electronic supplementary material:**

The online version of this article (doi:10.1007/s11306-016-1101-y) contains supplementary material, which is available to authorized users.

## Introduction

^1^H NMR is widely used for the metabolomic analysis of biofluids, as it provides quantitative, structural information on a wide range of metabolites, in a non-destructive and highly reproducible manner (Nicholson and Wilson 1989; Wishart 2008; Zhang et al. 2010). However, metabolite chemical shifts are sensitive to the chemical environment and subtle matrix effects, such as differences in pH and ionic strength, generally lead to inter-sample peak position variation (Weljie et al. 2006).

Urine is a popular biofluid used for metabolomic investigations, as it consists of various metabolites that can provide insight into a number of metabolic processes and disease states (Lindon et al. 1999). However, differences in its other components (such as urea, salts and other ions) are common, and result in often large variations in metabolite peak chemical shifts (Lindon et al. 1999). The main metal ions present in urine are sodium, potassium, and the divalent ions calcium and magnesium, however it is the divalent ions that are the main contributors (after pH) to peak chemical shift variability (Ackerman et al. 1996; Lindon et al. 2007; Yang et al. 2008).

A number of studies have investigated different sample preparation techniques in order to limit the pH and metal ion dependent NMR peak position variation. Buffers are commonly added to urine samples to control the pH variation of different samples. Lauridsen et al. (Lauridsen et al. 2007) recommended a minimum final concentration of 0.3 M for normal urine, and 1 M for concentrated urine samples. Xiao et al. (2009) recommended using 1.5 M buffer solutions in a 1:10 volume ratio of urine. While the use of a strong buffer may limit many metabolite peaks chemical shifts, a small number of metabolites, such as citrate and histidine, still exhibit strong inter-sample chemical shift variations (Lindon et al. 1999). In addition, very salty samples reduce NMR sensitivity, especially for cryogenically cooled probes. An alternative approach to minimise pH-dependent shifts is to move the sample pH to extreme values away from metabolite p*K*_a_ values and towards the acid/base chemical shift limits (Beneduci et al. 2011; Lehnert and Hunkler 1986; Sze and Jardetzky 1994; Wevers et al. 1999). A potential problem with this approach however may be the degradation of pH sensitive metabolites through hydrolysis or redox reactions.

A number of approaches have been proposed to remove the divalent metal ions from urine samples and therefore remove their influence on metabolite peak chemical shifts. Chelation of the divalent metals with EDTA has been used with promising results (Asiago et al. 2008; Ross et al. 2007). However, the use of EDTA introduces a number of ^1^H NMR signals for the free form as well as the Ca- and Mg-bound chelate forms. Deuterated EDTA is available, but this is quite expensive. Studies using biological ^31^P NMR have often used ion-exchange resins (such as Chelex-100) to remove paramagnetic metal ions, and some early work in ^1^H NMR metabolic profiling also used these to remove metal ions (Briceño et al. 2006; Cade-Menun and Preston 1996; Fan et al. 1997, 2001). Another proposed method for the removal of calcium and magnesium is the formation of insoluble fluoride salts of the metals by the addition of NaF or KF (Jiang et al. 2012). The metal precipitates can then be removed by centrifugation of the samples; however, this method may not be suitable for all metals and a combined approach with chelation may prove to be optimal (Jiang et al. 2012).

Despite the attempts to limit the metabolite peak chemical shifts, NMR spectra often require extensive pre-processing prior to chemometric analysis. A number of analytical strategies have been adopted to overcome shifting NMR peaks, such as spectral binning, automated alignment algorithms, or deconvolution methods that match metabolite standards to the NMR spectra (Alm et al. 2009; Anderson et al. 2011; Hao et al. 2012; Liebeke et al. 2013; Veselkov et al. 2009; Weljie et al. 2006). However each of these methods comes with certain drawbacks. For example, spectral binning results in loss of information and lower resolution, and still does not solve the problem of correspondence of peaks near bin boundaries; peak alignment strategies can introduce artefacts and are not suitable for the (common) situation where adjacent peaks cross over in frequency between samples; and successful peak deconvolution may require a lot of user input to get the best quality results.

Part of the problem lies in not knowing the extent of the shifts for individual peaks. Different metabolite protons have different chemical shift ranges and sensitivity to varying sample properties, and therefore global methods to account for peak shifts cannot be applied. A popular commercial peak-fitting software package, NMR Suite (Chenomx, Edmonton, Canada), provides chemical shift ranges of individual peaks and multiplets for common pH range values, and this assists the user to identify unknown metabolites; however this information is not freely available, and also does not account for shifts caused by other ions.

The pH dependency on NMR chemical shifts is not always detrimental: this phenomenon has been used to measure titration curves of various metabolites (Fan 1996). When a monobasic ligand (L) becomes protonated (HL), the change in the local electron density affects the chemical shift for certain nuclei (Szakács et al. 2004a). Since the protonation event occurs effectively instantaneously on the NMR time scale, the observed chemical shift (δ_obs_) is actually a weighted average of the limiting chemical shifts of the unprotonated (δ_L_) and the protonated (δ_HL_) states of the molecule (Ackerman et al. 1996; Szakács et al. 2004a). The weighting factors correspond to the pH-dependent mole fractions of L and HL, which can be expressed in terms of the actual pH and the acid dissociation constant *K*_a_ of the molecule as described by the Henderson-Hasselbalch equation (Ackerman et al. 1996; Szakács et al. 2004a). Therefore the observed chemical shift can be expressed as Eq. (1) (Ackerman et al. 1996), and the nonlinear fit of a molecule’s ^1^H NMR titration curve to this equation reveals the acid and base limiting chemical shifts δ_HL_ and δ_L_ respectively, as well as the acid dissociation constant *K*_a_ for that molecule.

1 $$\updelta_{\text{obs}} = \frac{{\updelta_{\text{L}} + \updelta_{\text{HL}} \left( {10^{{\left( {{\text{pH}} - {\text{p}}K_{\text{a}} } \right)}} } \right)}}{{1 + 10^{{\left( {{\text{pH}} - {\text{p}}K_{\text{a}} } \right)}} }}$$

For multibasic ligands (H*n*L), where *n* is the number of protonation sites within the metabolite, Eq. (1) can be extended to Eq. (2) (Szakács et al. 2004a).

2 $$\updelta_{\text{obs}} = \frac{{\updelta_{\text{L}} + \mathop \sum \nolimits_{i = 1}^{n} \updelta_{{{\text{H}}i{\text{L}}}} 10^{{\left( {\mathop \sum \nolimits_{{{\text{j}} = {\text{n}} - {\text{i}} + 1}}^{\text{n}} pK_{\text{j}} } \right) - {\text{ipH}}}} }}{{1 + \mathop \sum \nolimits_{k = 1}^{n} 10^{{\left( {\mathop \sum \nolimits_{l = n - k + 1}^{n} {\text{p}}K_{l} } \right) - k{\text{pH}}}} }}$$

This equation is much more complex than for the monobasic ligand, due to interaction between protons bound at different binding sites as well as the statistics of proton binding (Onufriev et al. 2001). Three different equilibrium constants—macroscopic, microscopic and quasisite—have been described for multibasic ligands (Onufriev et al. 2001; Ullmann 2003). The nonlinear fit of an NMR titration curve to Eq. 2 gives the macroconstants, but for molecules with multiple protonation sites with similar p*K*_a_ values, these macrospecies are actually mixtures of microspecies that hold identical numbers of protons but differ in the site of protonation (Ullmann 2003). Thus, the macroconstants only refer to the stoichiometry of proton binding, not the site of protonation, and a ligand with *n* protonation sites has 2^*n*^ microstates. It is also possible to characterise the titration curve of a multibasic ligand with *n* protonation sites as a sum of *N* noninteracting so called quasisites; see Onufriev et al. for a more in-depth treatment (Onufriev et al. 2001).

Many studies that have used NMR to investigate pH titration curves have focused on solutions of single metabolites (Bezençon et al. 2014) or mixtures of a small number of metabolites (Xiao et al. 2009), whereas in metabolomics investigations complex biological matrices are analysed. Here, we have characterised the pH- and ion-dependent (Na^+^, K^+^, Ca^2+^, Mg^2+^) chemical shift changes that occur in the ^1^H NMR spectra of urine. Knowledge of the acid, base and metal ion dependent chemical shift limits of the main metabolites in a complex biological mixture will be important for metabolite assignments and will provide information that we hope may prove valuable in the future to help with designing alignment algorithms and automatic peak detection methods.

## Materials and methods

Urine samples from five different individuals were collected in 1 day between 10 am and 3 pm, and placed on ice. The collected samples were pooled, and then frozen at −80 °C in aliquots of approximately 150 ml. Prior to the NMR experiments, urine was passed through a column of Chelex-100 ion exchange resin (BioRad) to remove the majority of Ca^2+^ and Mg^2+^ ions, and the pH indicator standards imidazole, formate, Tris and piperazine were added to a final concentration of 1 mM, 1 mM, 0.25 mM and 0.25 mM, respectively. We measured the main metal ion concentrations in urine (Ca^2+^, Mg^2+^, Na^+^ and K^+^) with ion selective electrodes and pH measurements were performed with a glass electrode with inbuilt temperature sensor (Fisher Scientific).

For the pH titration experiment, two volumes of Chelex-treated urine (200 ml) were treated dropwise with either 1 M HCl or 1 M NaOH while stirring. Samples were taken for NMR (400 μl) at 0.2 pH unit intervals and were prepared for NMR with addition of H_2_O (180 μl) and ^2^H_2_O (20 μl) containing 4,4-dimethyl-4-silapentane-1-sulfonic acid-^2^H_6_ (DSS) to give a final concentration of 0.1 mM. For the ion titration experiment, Chelex-treated urine (400 μl), was supplemented with H_2_O containing various concentrations of CaCl_2_, MgCl_2_, NaCl or KCl, ranging from 0 to 1 M, and D_2_O (20 μl) containing DSS to give a final concentration of 0.1 mM. NMR samples were centrifuged for 5 min at 13,000 rpm and 550 μl was transferred to a 5 mm NMR tube. Spectra were acquired on a Bruker Avance DRX600 NMR spectrometer (Bruker BioSpin, Rheinstetten, Germany), with ^1^H frequency of 600 MHz, and a 5 mm inverse probe at a constant temperature of 300 K. Samples were introduced with an automatic sampler and spectra were acquired following the procedure described by Beckonert et al. (2007). Briefly, a one-dimensional NOESY sequence was used for water suppression; data were acquired into 64 K data points over a spectral width of 12 kHz, with 8 dummy scans and 128 scans per sample. Spectra were processed in iNMR 3.4 (Nucleomatica, Molfetta, Italy). Fourier transform of the free-induction decay was applied with a line broadening of 0.5 Hz. Spectra were manually phased and automated first order baseline correction was applied. Metabolites were assigned at Metabolomics Standards Initiative (MSI) level 2 using the Chenomx NMR Suite 5.1 (Chenomx, Inc., Edmonton, Alberta, Canada). Metabolite peak positions from the different samples relative to DSS were obtained using MATLAB scripts written in-house by Dr Gregory Tredwell, and appropriate chemical shifts were determined for multiplets. A version of the scripts for peak picking and spline fits are part of the BATMAN project (batman.r-forge-project.org) (Liebeke et al. 2013). The observed chemical shifts of the various metabolite peaks were modelled with respect to pH with the general formula (Eq. 2) for multibasic acids (H_n_L), using the nlinfit function within MATLAB. The number of sites was assumed from the chemical structure. This enabled the estimation of p*K*_a_ values and acid and base chemical shift limits for individual metabolite peaks.

## Results and discussion

In order to systematically characterise the pH and ionic variation in a biologically relevant sample matrix, we collected a urine sample from five different individuals and pooled it to obtain a single large-volume representative human urine sample. Our aim was to manipulate the pH of the urine over a wide pH range and measure the resultant peak variability with ^1^H NMR. It is likely that the large pH changes would alter metal ion concentrations in the urine, which could themselves interfere with the metabolite peak chemical shifts. To limit the effect of these changes, the main divalent metal ions, such as Ca^2+^ and Mg^2+^, were removed from the urine sample using Chelex ion exchange resin before the pH adjustment step. The treatment reduced the concentrations of Ca^2+^ and Mg^2+^, without compromising the metabolic composition of the urine (Supplementary Fig. 1, Supplementary Table 1). While the ion exchange resin was successful in removing the divalent ions, K^+^ concentrations were only slightly reduced by the resin treatment, and Na^+^ concentrations were slightly increased as they were displaced from the resin by the divalent ions. This was unfortunate as the sample ionic strength has the potential to alter certain metabolite p*K*_a_ values and acid/base chemical shift limits (Xiao et al. 2009). However, as the increase in Na^+^ from the Chelex resin was relatively small compared with commonly used buffer concentrations (Lauridsen et al. 2007; Xiao et al. 2009), and as the main ionic contributors to NMR peak shift variations are the divalent ions Ca^2+^ and Mg^2+^ (Ackerman et al. 1996; Lindon et al. 2007, 2011; Yang et al. 2008), we felt that this was a suitable baseline urine sample to continue our investigations.

**Fig. 1:**
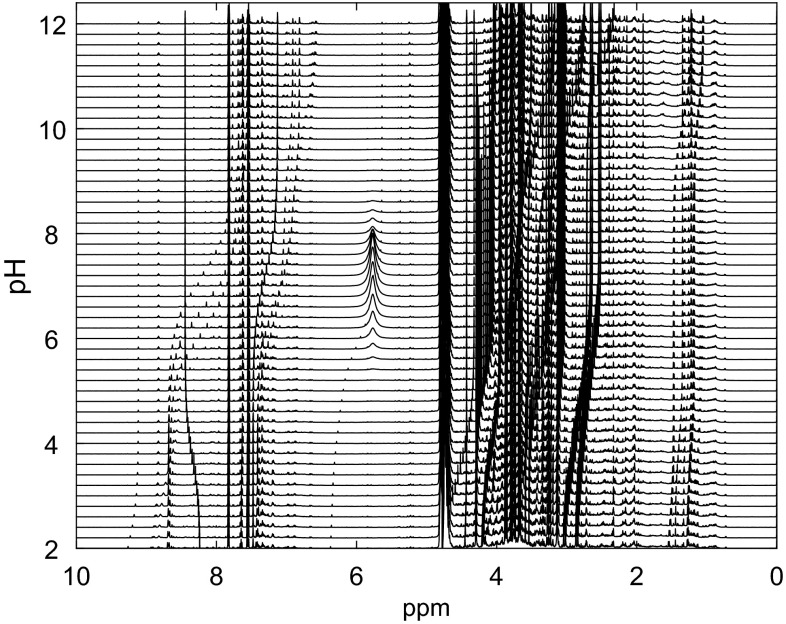
^1^H NMR spectra of human urine with pH adjusted from 2 (*bottom*) to 12 (*top*). Adjacent spectra are 0.2 pH units apart

The ^1^H NMR data for a single urine sample at 51 pH values over the range of 2–12 are shown in Fig. 1. Large chemical shift changes occur for a number of metabolite peaks. Individual peak positions were picked across all samples with the help of MATLAB scripts written in-house. In total the shifts for 163 individual peaks were obtained, corresponding to 53 multiplets from 33 identified metabolites and 65 currently unassigned peaks. As Eqs. 1 and 2 assume that the reference chemical shift does not change, chemical shifts of the metabolite peaks were determined relative to DSS. DSS is a salt of a strong acid, and therefore its ionization state and hence chemical shift is stable over the pH range 2–12 (Szakács et al. 2004b). The nonlinear fitting of peak or multiplet pH dependent chemical shifts with respect to Eq. 2 was performed with the nlinfit MATLAB script, and p*K*_a_ values and acid/base limits for 33 identified metabolites were modelled (Table 1). The modelled p*K*_a_ values were in fairly good agreement with literature values (Lundblad and Macdonald 2010), though some metabolites did show some differences. These are likely to be due to ionic interactions with sodium ions that could not be removed from the samples, and matrix effects of the complex biofluid. Characterising these effects was the goal of this study, as these data would then more accurately represent future metabolite profiling experiments.

**Table 1:** Modelled p*K*_a_ and acid and base limits for 33 metabolites from human urine. Literature p*K*_a_ values sourced from (Lundblad and Macdonald 2010)

Metabolite	Literature pKa values			Modelled pKa values			Modelled acid and base limits			
Methylnicotinamide				3.605			9.265	9.269		
Hydroxyisobutyrate				3.813			1.347	1.451		
Hydroxyisovalerate				4.324			1.260	1.332		
Methyl2Oxovalerate				3.843			1.093	1.135		
Acetate	4.760			4.578			1.910	2.089		
Alanine	2.340	9.690		2.394	9.979		1.211	1.472	1.572	
Allantoin	8.960			8.752			5.360	5.384		
Citrate	3.090	4.750	5.410	2.938	4.268	5.504	2.528	2.683	2.774	2.866
Citrate	3.090	4.750	5.410	3.758	5.275	6.209	2.646	2.668	2.785	2.970
Creatinine	4.840	9.200		2.787	4.900		3.033	3.134	3.136	
Creatinine	4.840	9.200		3.284	4.921		4.043	4.286	4.296	
Formate	3.770			3.555			8.448	8.234		
Glucose	12.430			12.067			5.273	5.228		
Hippurate	3.620			3.482			3.960	4.200		
Hippurate	3.620			3.417			7.541	7.552		
Hippurate	3.620			3.450			7.627	7.649		
Hippurate	3.620			3.300			7.824	7.830		
Hippurate	3.620			3.468			8.512	8.839		
Histidine	1.820	6.000	9.170	6.397	7.346	8.939	6.815	6.924	7.039	7.414
Histidine	1.820	6.000	9.170	5.938	6.966	9.355	7.663	7.727	7.914	8.664
Imidazole	6.950			7.007			7.129	7.477		
Imidazole	6.950			7.027			7.768	8.682		
Isoleucine	2.320	9.760		2.385	9.842		0.902	1.001	1.037	
Lactate	3.860			3.678			1.320	1.419		
Leucine	2.330	9.740		2.436	9.487		0.932	0.957	0.974	
Mannitol	13.500			2.602			3.673	3.674		
Mannitol	13.500			12.084			3.792	3.797		
Mannitol	13.500			11.441			3.857	3.864		
MethylSuccinate	4.130	5.640		3.238	4.569		1.062	1.118	1.197	
Phenylalanine	1.830	9.130		3.737	6.600		7.349	7.351	7.343	
Phenylalanine	1.830	9.130		4.365	10.110		7.413	7.413	7.412	
Piperazine	5.560	9.830		5.779	10.116		2.739	3.116	3.590	
TMethylHistidine	1.690	6.480	8.850	1.797	5.889	9.389	6.873	6.972	7.356	7.431
TMethylHistidine	1.690	6.480	8.850	5.853	7.301	9.455	7.510	7.568	7.648	8.631
TTMethylHistidine	1.690	6.480	8.850	6.378	7.313	8.942	3.609	3.646	3.698	3.862
TTMethylHistidine	1.690	6.480	8.850	1.813	6.056	9.345	6.909	7.036	7.390	7.460
TTMethylHistidine	1.690	6.480	8.850	6.402	7.379	8.937	7.562	7.668	7.882	8.686
Tartrate	2.890	4.160		3.045	4.039		4.322	4.472	4.720	
Taurine	1.500	8.740		7.206	9.096		3.008	3.412	3.425	
Threonine	2.630	10.430		2.254	9.201		1.194	1.322	1.361	
Trigonelline	1.860			2.153			4.429	4.469		
Trigonelline	1.860			2.085			8.073	8.180		
Trigonelline	1.860			2.093			8.822	8.976		
Trigonelline	1.860			2.007			8.834	9.030		
Trigonelline	1.860			2.037			9.115	9.398		
Tris	8.300			8.439			3.508	3.734		
Tryptophan	2.380	9.390		3.694	5.491		7.719	7.713	7.708	
Tyrosine	2.200	9.110	10.070	4.132	10.208	10.807	6.576	6.622	6.852	6.885
Tyrosine	2.200	9.110	10.070	4.081	10.270	11.269	7.000	7.003	7.156	7.207
Valine	2.320	9.620		2.342	9.505		0.906	0.981	1.050	
Valine	2.320	9.620		8.609	10.199		0.922	0.991	1.060	
Xylose	12.150			12.369			5.284	5.190		
transAconitate	2.800	4.460		3.585	5.404		6.574	6.745	7.007	

**Fig. 2:**
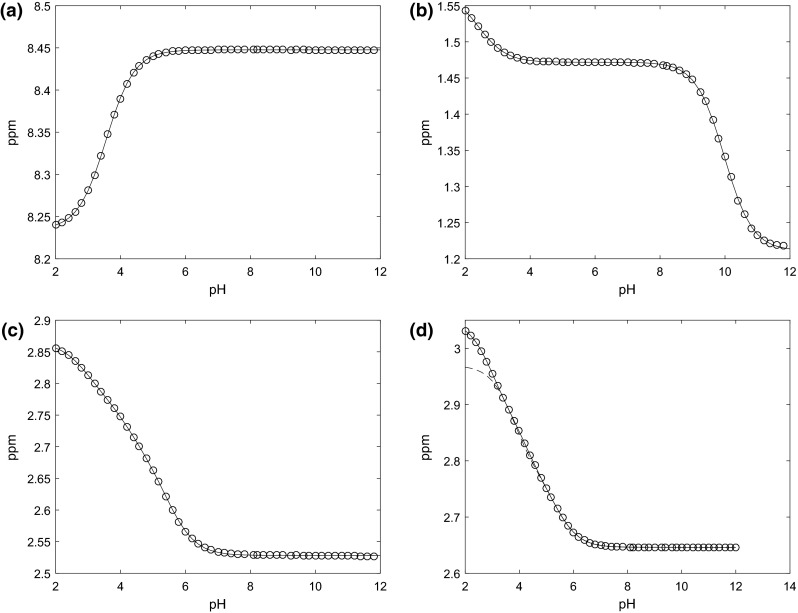
Measured chemical shift changes (*open circle*) for Formate (**a**), Alanine (**b**) and Citrate (**c**) peaks are shown with non-linear fits to the one, two and three protonation site forms of Eq. (2), respectively (*line*). A second Citrate peak (**d**) is shown with a nonlinear fit to the three protonation site form of Eq. (2) (*dotted line*) versus the four protonation site form of Eq. (2) (*line*)

An example of these model fits for one, two and three site protonation models of formate, alanine, and citrate respectively, is shown in Fig. 2. The two pairs of geminal methylene protons of citrate produce a strongly coupled AB spin system in ^1^H NMR spectra (Moore and Sillerud 1994) and the two A and B chemical shifts are shown in Fig. 2c, d. The models closely agree with measured chemical shifts in most cases, although one of the citrate peaks (Fig. 2d) was better fitted by a four site protonation model. It is not clear why this was the case. As previously described, the urea amide peak at 5.7 ppm, at both high and low pH values is seen to gradually disappear due to enhanced rates of proton exchange at these pH values (Xiao et al. 2009).

**Fig. 3:**
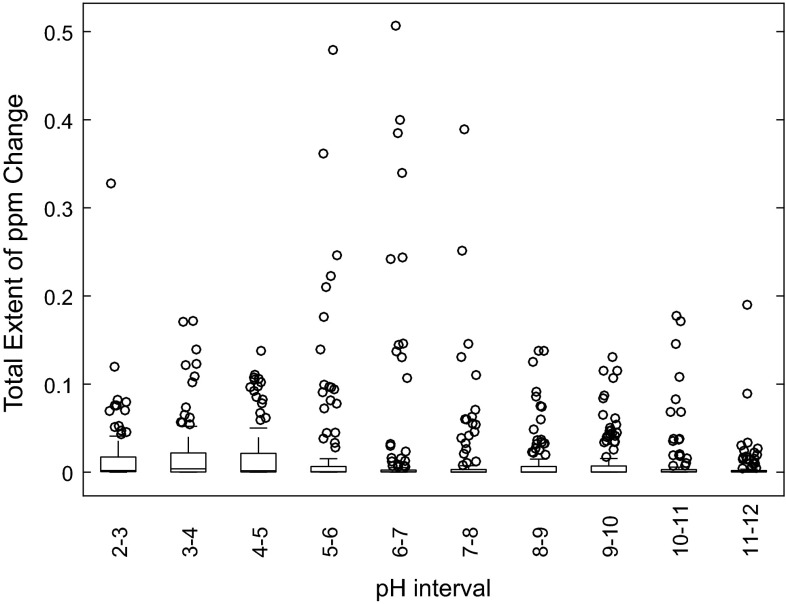
Total extent of ppm change for 163 ^1^H NMR peaks in human urine over the range of pH 2–12 in one pH unit intervals

NMR samples are generally buffered to neutral or near-neutral pH using phosphate buffer, but there is no absolute need for this, and some studies have chosen more extreme sample pH endpoints in order to move away from the p*K*_a_ values of highly shifting metabolites (Beneduci et al. 2011; Lehnert and Hunkler 1986; Sze and Jardetzky 1994; Wevers et al. 1999). If the chemical shift differences of metabolites between spectra can be minimised, the spectral quality would be increased and hence also the data processing simplified. For a mixture of nine urinary metabolites, Xiao et al. (Xiao et al. 2009) found the pH range of 7.1–7.7 was optimal for most of these metabolites. Figure 3 shows the extent of 163 metabolite peak chemical shift changes over single pH units for the pH range 2–12. The pH intervals 5–6, 6–7 and 7–8 have some of the lowest median peak chemical shift changes of 0.0006, 0.0003 and 0.0003 ppm respectively; however, these pH ranges also show some of the largest chemical shift changes for a small number of metabolite peaks. It is clear though, for all the pH ranges measured in this study, a small number of metabolite peaks still have significant chemical shift variability, which is likely to present problems for data processing no matter the chosen sample pH. These data will therefore be invaluable to the design of more sophisticated data processing methods in order to account for the metabolite peak chemical shift variability at a given sample pH.

Our current data indicate that it might be worth considering NMR buffers with higher p*K*_a_ values, as the pH range from 8 to 9 also had a low median peak chemical shift change of 0.0003 ppm, but also showed a decreased range for the outlying highly shifting peaks, and the pH range 11–12 had the lowest average chemical shift difference (Fig. 3). Working at high pH values would also have the advantage that the signal from the urea peak would be removed or have reduced intensity. There would of course also be some disadvantages to such an approach: it is likely that different metabolite classes (e.g. nucleotides) would have reduced stability at higher pH values. However, there is no reason why adjustment to high pH values would not be possible so long as studies were kept internally consistent (Sze and Jardetzky 1994).

**Fig. 4:**
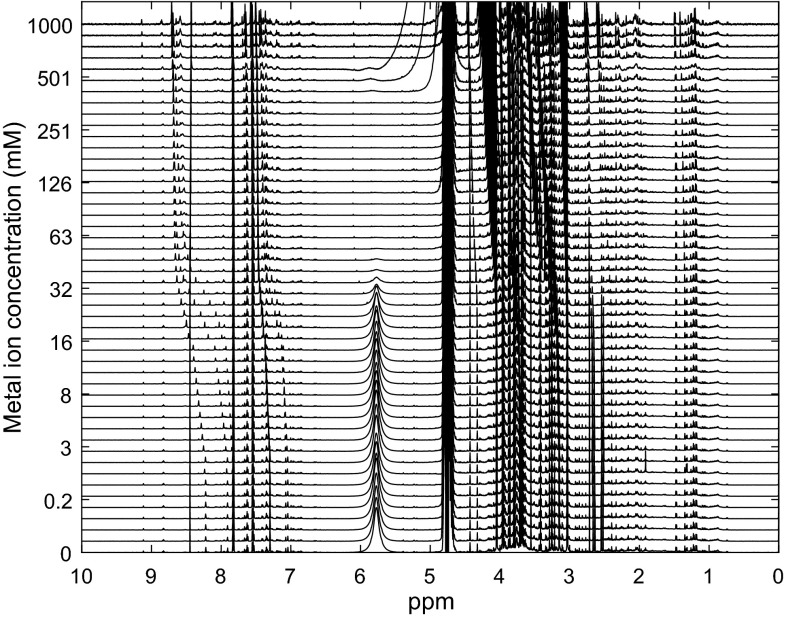
^1^H NMR spectra of human urine with Ca^2+^ adjusted from 0.01 (*bottom*) to 1000 mM (*top*) CaCl_2_

**Fig. 5:**
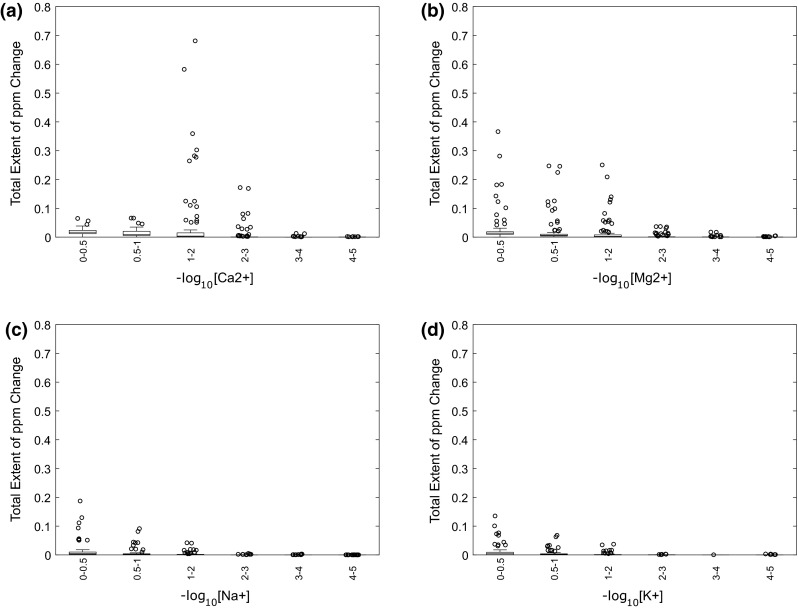
Chemical shift variations in human urine for 33 identified metabolites when adjusted with CaCl_2_ (**a**), MgCl_2_ (**b**), NaCl (**c**) and KCl from 0.01 to 1 M in −log_10_[metal ion] units

The effects of the main metal ions present in human urine, Na^+^, K^+^, Ca^2+^ and Mg^2+^, on metabolite chemical shift changes were also investigated. We added the metals (as chloride salts) to the ion-exchange-resin-treated urine sample, to final concentrations ranging from 0.01 mM to 1 M. As expected the divalent metal ions, in particular Ca^2+^, induced the most prominent chemical shift changes (Fig. 4, Supplementary Fig. 2), followed by Mg^2+^, and then the monovalent metal ions Na^+^ and K^+^. Concentrations of Ca^2+^ ions between 10 and 100 mM showed the largest chemical shifts, with little further change in peak chemical shift at higher concentrations (Fig. 5). Normal concentrations for urinary calcium are between 0.225 and 9.47 mM for males and between 0.125 and 8.92 mM for females, though this is largely dependent of diet (Wu 2006), while Jiang et al. (Jiang et al. 2012) measured an average Ca^2+^ concentration in rat, mouse and human urine samples to be 7.4, 1 and 0.9 mM respectively. The magnitudes of the Ca^2+^-induced peak shifts correlated well to those induced by decreasing the pH, with a maximum peak shift of 0.99 ppm (Supplementary Table 2, Supplementary Data). In contrast, concentrations of Mg^2+^ ions between 10 and 100 mM showed only moderate peak shifts, but these continued for concentrations up to 1 M, and the magnitude of Mg^2+^-induced chemical shift changes correlated well with acid-induced changes with a maximum of 0.76 ppm (Fig. 5). For the monovalent ions, concentrations between 331 mM and 1 M showed peak shifts >0.1 ppm for only a small number of metabolites, with maximum peak chemical shift changes of 0.33 and 0.24 ppm for Na^+^ and K^+^, respectively (Fig. 5). As seen with the pH-induced changes on peak chemical shifts, the largest metal ion-induced peak variations were for the azole-class metabolites histidine, and methyl-histidines. Histidine’s affinity for a number of metal ions is well known (Maley and Mellor 1949).

A complication of attempting to characterise the metal ion effects on metabolite peak shifts in the ^1^H NMR spectra of human urine was the increase in pH due to displacement of H^+^ ions by the metal ions (Supplementary Fig. 3). This effect was more pronounced for the divalent ions—especially Ca^2+^ and to a lesser extent Mg^2+^. We considered using a buffered urine sample for this test, to try and reduce the decrease in pH, but decided against it as interaction of the buffer itself with the different metal ions could not be ruled out. Good’s buffers, a class of zwitterionic *N*-substituted aminosulfonic acid buffers, have been considered to have weak metal complexation properties; however some reports suggest that many of these buffers in fact strongly bind metals (Mash et al. 2003). Furthermore, the addition of these buffers would introduce interfering peaks in the ^1^H NMR spectra, potentially overlapping with important metabolite signals, as well as introducing additional counter ions. We therefore considered it to be the ‘least flawed’ approach to work with the unbuffered urine, and attempt to correct to some extent based on our knowledge of the pH-responsiveness of individual metabolite resonances. For the metal ion experiments, when the metal ion induced peak shifts are plotted with respect to the changing pH, it is clear that the metal ions alter peak chemical shifts independent of the pH (Supplementary Fig. 4). Not only are differences between the monovalent and divalent metal ions on peak chemical shift changes evident, but also differences between Ca^2+^ and Mg^2+^ ions can be observed. While we have yet to develop a model to predict metabolite chemical shift changes based on metal ion concentrations, these data will still be useful baseline data.

## Conclusion

The pH-dependent chemical shift changes for a large number of metabolite peaks were modelled effectively using a modified equation based on the Henderson–Hasselbalch equation (Eq. 2). From these models we have obtained p*K*_a_ values and acid/base peak chemical shift limits for 33 identified metabolites from human urine, as well as provided chemical shift data for a further 65 unassigned metabolite peaks for a wide pH range of 2–12. Furthermore, we have characterised the effects of the four metal ions commonly present in human urine, Na^+^, K^+^, Ca^2+^ and Mg^2+^ over a wide concentration range to better understand the ionic effects on metabolite peak chemical shift variability. Knowledge of the modelled p*K*_a_ and acid/base limits will give confidence to metabolite peak positions for a given sample pH and together these data will be valuable for the development of automated metabolite alignment and identification algorithms for ^1^H NMR spectra.

## Electronic supplementary material

Below is the link to the electronic supplementary material.

Supplementary material 1 (ZIP 15695 kb)

Supplementary material 2 (DOCX 12 kb)
