# Supplementary material for: Modelling the acid/base 1H NMR chemical shift limits of metabolites in human urine
Source: Metabolomics. 2016 Sep 15;12(10):152. doi: 10.1007/s11306-016-1101-y (PMC5025509; doi:10.1007/s11306-016-1101-y)
Supplement: Supplementary file 2 — Supplementary material 2 (DOCX 12 kb) [file 11306_2016_1101_MOESM2_ESM.docx]

## Supplementary Figure and Table Legends

Supplementary Table 1 Metal ion concentrations in the human urine sample before and after treatment with Chelex resin.

Supplementary Figure 1 ^1^H NMR spectra of human urine before (red) and after (blue) treating with Chelex resin.

Supplementary Figure 2 ^1^H NMR spectra of human urine adjusted with MgCl_2_ (a), NaCl (b) and KCl (c) from 0.01mM (bottom) to 1M (top).

Supplementary Table 2 Spearman correlations of the magnitude of acid induced peak shifts (pH 2-7) with the metal ion induced peak shifts (0 - 1M) in the ^1^H NMR of human urine.

Supplementary Figure 3 The pH changes induced in human urine by the addition of CaCl_2_, MgCl_2_, NaCl and KCl from 0.01 mM to 1000 mM.

Supplementary Figure 4 The metal ion induced peak shift changes for citrate (a, b), histidine (c, d) and hydroxyisovalerare (e) plotted with respect to pH changes in the metal ion addition experiments. The orange arrows represent the low metal concentration points.

Supplementary Figure 5 ^1^H NMR spectra of human urine with pH adjusted from 2 (bottom) to 12 (top). Adjacent spectra are 0.2 pH units apart. This view is an expansion of Figure 1 for the 4.5 to 0.1 ppm range

Supplementary Figure 6 ^1^H NMR spectra of human urine with CaCl_2_ (a), MgCl_2_ (b), NaCl (c) and KCl (d) from 0.01 mM (bottom) to 1000 mM (top). This view is an expansion of Figure 4 and Supplementary Figure 2 for the 4.5 to 0.1 ppm range.
